# Nontuberculous Mycobacterial Musculoskeletal Infection Cases from a Tertiary Referral Center, Colorado, USA

**DOI:** 10.3201/eid2506.181041

**Published:** 2019-06

**Authors:** Noah Goldstein, J. Benjamin St. Clair, Shannon H. Kasperbauer, Charles L. Daley, Bennie Lindeque

**Affiliations:** University of Colorado, Aurora, Colorado, USA (N. Goldstein, J.B. St. Clair, S.H. Kasperbauer, B. Lindeque);; University of Chicago, Chicago, Illinois, USA (J.B. St. Clair);; National Jewish Health, Denver, Colorado, USA (S.H. Kasperbauer, C.L. Daley)

**Keywords:** nontuberculous mycobacteria, *Mycobacterium*, musculoskeletal diseases, prosthesis-related infections, osteomyelitis, infectious arthritis, tuberculosis and other mycobacteria, antimicrobial resistance, Colorado, United States

## Abstract

Nontuberculous mycobacteria represent an uncommon but important cause of infection of the musculoskeletal system. Such infections require aggressive medical and surgical treatment, and cases are often complicated by delayed diagnosis. We retrospectively reviewed all 14 nonspinal cases of nontuberculous mycobacterial musculoskeletal infections treated over 6 years by orthopedic surgeons at a university-affiliated tertiary referral center. All patients required multiple antimicrobial agents along with aggressive surgical treatment; 13 of 14 patients ultimately achieved cure. Four patients required amputation to control the infection. Half these patients were immunosuppressed by medications or other medical illness when they sought care at the referral center. Six cases involved joint prostheses; all ultimately required hardware removal and placement of an antimicrobial spacer for eradication of infection. Our findings highlight the importance of vigilance for nontuberculous mycobacterial musculoskeletal infection, particularly in patients who are immunosuppressed or have a history of musculoskeletal surgery.

## Abstract 2

Early identification and aggressive treatment are crucial for patients with these infections.

Nontuberculous mycobacteria (NTM) are opportunistic pathogens found in soil and water and are the sources of an increasing number of infections and illness (*1*,*2*). Multiple recent studies have recognized this temporal trend of increasing annual prevalence of NTM disease; current prevalence is estimated at 2.6%–10% annually (*1*–*4*). NTM cause a broad range of clinical manifestations, depending on the immune competency of the host. Pulmonary disease is most common and is often seen in immunocompetent hosts. It is difficult to estimate the prevalence of extrapulmonary NTM infection in the United States because no system of national surveillance and reporting exists as for tuberculosis. Reported laboratory isolation data indicate that extrapulmonary disease may comprise up to one quarter of all NTM infections, although the best estimates are based on data that are 1–3 decades old. Data from the mid-1990s estimated the overall US incidence of NTM infection at 1.0%–1.8%, with ≈5% of isolates reported to the Centers for Disease Control and Infection at that time being of extrapulmonary origin. A population-based record review in Oregon that used data gathered during 2005–2006 found ≈22% of isolates originating from nonpulmonary specimens. Data from North Carolina gathered during 2006–2010 showed 16%–23% of total isolates originating from extrapulmonary specimens (*5*–*7*). Skin and soft tissue infections represent most extrapulmonary NTM disease, whereas bone and joint disease is less common. With increasing immunosuppression, NTM is more likely to manifest as disseminated disease, possibly including bone and joint involvement.

Previous case reports and series of NTM musculoskeletal infections have focused on the demographics, diagnosis, and treatment of patients with these difficult-to-manage infections (*8*–*24*). Patients were often immunocompetent persons who contracted an NTM disease after direct inoculation via traumatic skin puncture (*25*–*28*). In a series of 29 cases from South Korea, all patients required surgical intervention in addition to antimicrobial drugs; 3 patients failed to respond despite aggressive treatment, and an additional 4 were lost to follow-up. Of note, only 20 of the patients in this series received NTM-specific antimicrobial therapy; the remainder were treated with empirical antimicrobial therapy. A series of 28 cases of *M. marinum* musculoskeletal infections found that 93% of cases involved fingers or hands and 87% were related to aquatic exposure; two thirds progressed from cutaneous to invasive infection. Most cases were managed with multiple antimycobacterial agents in addition to surgery, and the series reported a 75% cure rate and 25% loss to follow-up. A study of 8 cases of prosthetic joint infection (PJI) caused by rapidly growing mycobacteria found that the median interval from prosthesis implantation to infection was 312 weeks and that all patients required either resection and explantation of infected prostheses or chronic suppressive antimicrobial therapy.

In these series, infections occurred throughout the musculoskeletal system but were particularly common in the hands and spine. Identification of NTM as the causative agent was often delayed due to the indolent course of the infection and lack of appropriate diagnostic testing. Once identified, the infections could be treated effectively with a combination of debridement and conventional antimycobacterial chemotherapy.

We reviewed all cases of nonspinal NTM musculoskeletal infection treated at the University of Colorado Hospital (UCH; Aurora, CO, USA) over a 6-year period by a multidisciplinary team of providers at UCH and National Jewish Health (NJH; Denver, CO, USA), a renowned center for mycobacterial research and treatment that receives referrals for difficult-to-treat NTM infections from across the United States. Our aim was to describe the clinical characteristics and treatment outcomes of patients with NTM musculoskeletal infections.

## Materials and Methods

### Study Design

We performed a retrospective study of patients with nonspinal NTM musculoskeletal infections treated at UCH during 2009–2015. The management of each case was directed by an orthopedic surgeon and infectious disease physicians at UCH and NJH. For patients with positive culture results at UCH, mycobacterial speciation and susceptibility data were identified by ARUP Laboratories (https://www.aruplab.com) using DNA sequencing or matrix-assisted laser desorption/ionization time-of-flight mass spectrometry.

### Case Ascertainment

We identified all cases of nonspinal NTM musculoskeletal infections treated by orthopedic surgeons at UCH during the study period using a registry of NTM musculoskeletal infections. We omitted spinal cases because they are handled by a different department at UCH and so were not part of the surgeons’ personal registry. We reviewed the medical records of patients after approval from the Institutional Review Board.

### Data Collection

We performed our review of the medical records using a standardized case report form. We collected demographic characteristics and data on concurrent conditions, laboratory values, surgical intervention, antimycobacterial chemotherapy, and outcomes.

### Literature Review 

We searched the Medline database to identify case reports and reviews of NTM musculoskeletal infections. We used both the PubMed and Ovid access tools.

## Results

### Patient Characteristics

Of the 14 patients in our series, 7 were receiving immunosuppressive medications at arrival, and 5 of those had chronic autoimmune disease. Twelve patients were referred from NJH. No predisposing host factor was identified for 6 patients. (Table 1).

**Table 1:** Demographics of patients in retrospective study of nontuberculous mycobacterial musculoskeletal infections, Colorado, USA*

Patient no.	Age, y/sex	Immunocompromising conditions	Inciting or predisposing factor(s)	Time to symptom onset, mo	Reason care sought	Time from symptom onset to diagnosis, mo	Diagnostic procedure, source of microbiologic diagnosis
1	55/M	History of lymphoma, pseudogout, oral steroid use	Steroid injection, left ankle	UNK	Osteomyelitis, septic arthritis	12	Tissue sample from ankle debridement
2	53/F	None	Pedicure	0	Synovitis, osteomyelitis, cellulitis	4	UNK; diagnosis at another hospital
3	48/F	None	Second THA	UNK	PJI	UNK	Thigh fluid sampled during I&D
4	45/M	None	AKA	1	Draining wound at stump	2	Sample of fluid draining from AKA stump
5	64/F	None	Carpal tunnel release	0	Tenosynovitis, septic arthritis	8	Synovial fluid obtained during excision of synovitis
6	69/F	Diabetes mellitus	THA	6	PJI	9	Synovial biopsy obtained during explantation
7	23/F	Systemic lupus erythematosus, oral steroids, hydroxyl-chloroquine, mycophenolate mofetil	Avascular necrosis, ankle fusion	3	Septic arthritis, osteomyelitis, skin abscesses	1	UNK; diagnosis at another hospital
8	57/F	Polymyositis, oral steroids, azathioprine, rituximab, methotrexate	ACL repair, arthroscopy, steroid injection	UNK	Septic arthritis	UNK	Aspirate of left knee and right hip
9	76/M	Nonspecific autoimmune disease, oral steroids	Calcium pyrophosphate deposition disease, TKA	0	PJI	2	UNK; diagnosis at another hospital
10	58/M	None	Trauma, steroid injection	12	Septic arthritis	19	UNK; diagnosis at another hospital
11	77/F	Rheumatoid arthritis, prednisone, azathioprine	TKA	300	PJI	12	Synovial biopsy obtained during placement of antimicrobial spacer
12	83/M	Malnutrition, prednisone	TKA	168	PJI	8	Tissue obtained during TKA revision
13	49/F	None	TKA (revision)	14	PJI	2	UNK; diagnosis at another hospital
14	24/F	Crohn’s disease, infliximab	Failed IV	0	Cellulitis, abscess	7	Skin biopsy

*AKA, above-knee amputation; I&D, irrigation and debridement; PJI, prosthetic joint infection, THA, total hip arthroplasty; TKA, total knee arthroplasty; UNK, unknown.

### Clinical Presentation and Diagnosis

The patients had a wide array of sites of infection when they sought care at UCH (5 knee, 2 hip, 1 thigh, 1 hand, 1 foot, 1 elbow, 3 with disseminated disease at multiple sites). The diagnosis at arrival was most commonly PJI (n = 6); the next most common diagnoses included septic arthritis (n = 5), osteomyelitis (n = 3), tendon sheath infection (n = 2), skin abscess (n = 2), cellulitis (n = 2), and surgical-wound infection (n = 1). Several patients had complex local infections with multiple concurrent tissue diagnoses; case-patients 1, 7, and 8 had disseminated disease at multiple sites. Each patient had undergone >1 prior procedure at the site of infection, including 6 patients with infected prostheses, 4 with other surgical procedures, 2 with steroid injection, 1 with a failed intravenous (IV) placement, and 1 with a cosmetic pedicure. The median interval from symptom onset to diagnosis was 7.5 months (range 1–19 months). 

Of 10 patients tested, the median erythrocyte sedimentation rate (ESR) was above the upper limit of normal (ULN) in 7 patients (70%) at arrival (ULN 15 mm/h in men, 20 mm/h in women). C-reactive protein level was elevated in 4/14 patients (29%) (ULN 10.0 mg/L). Blood leukocyte count was elevated in 2/14 patients (14%) (ULN 10.1 × 10^4^ cells/μL). All 3 laboratory tests were within reference limits in 1 patient, and both C-reactive protein and leukocyte levels were within reference limits in an additional 2 patients without ESR data.

### PJIs

Six patients arrived with PJI at the knee (4 patients) and hip (2 patients). The interval from prosthesis implantation to symptom onset ranged from the immediate aftermath of implantation to 25 years postimplantation; median duration was 6 months. Infected hardware was removed in all patients with PJI; the median period from symptom onset to explantation was 8.5 months (range <3–16 months). After explantation, 2-stage revision was undertaken, first with implantation of a temporary articulating prosthesis prepared with 1 g of antimicrobial-loaded polymethylmethacrylate bone cement, with drug selection based on sensitivity testing. This preparation, called an antimicrobial spacer, was subsequently replaced with a permanent prosthesis after several months without evidence of recurrent infection. Medication-loaded bone cement was also used for patients requiring intramedullary nailing. Patients with PJI were also treated with amikacin-containing calcium sulfate beads implanted within the surgical bed (1 g of amikacin per 10 cc packet of calcium sulfate mixture). These antimicrobial beads were used at initial explantation or when the antimicrobial spacer was replaced with a permanent prosthesis at the discretion of the surgeon as needed to fill dead space from aggressive debridement or the infection itself. The precise amount of amikacin used is not always clearly reflected in the surgical record; however, in our series, use of up to 5 g of amikacin has been recorded. Amikacin is used for its activity against mycobacteria and its ability to integrate with the calcium sulfate material to form beads. Such amikacin-laden beads were also used in 2 other patients with extensive soft-tissue involvement. A seventh patient with septic arthritis of her native knee (case-patient 8) underwent a total knee arthroplasty that was insufficient to eradicate the infection and ultimately required subsequent 2-stage revision.

### Surgical Treatment

All patients required surgical treatment for control of infection, and all but 1 patient were ultimately cured by combined medical and surgical treatment. Seven patients had >1 procedure at outside hospitals before seeking treatment at UCH. Case-patients 4 and 5 required only a single surgical procedure at UCH for control of infection. Case-patients 6 and 12 underwent planned 2-stage revision and required no further surgeries. The remaining 10 case-patients had initial procedures at UCH that were intended to definitively treat infection but proved unsuccessful. Surgical treatment at UCH consisted of aggressive debridement of infected bone and soft tissue, as well as explantation of any infected hardware with 2-stage revision of infected prostheses. Four patients with infections involving the knee also required placement of intramedullary nails coated with antibiotic-loaded cement (case-patients 9, 10, 11, and 13). The median interval from initial diagnostic or therapeutic procedure to definitive surgical treatment was 14 months (range 4–52 months) in the patients who required multiple procedures. One patient with disseminated *M. abscessus* (case-patient 1) required a debridement of the femur, partial claviculectomy, synovectomy of an ankle and a knee, and a fasciectomy. The patient with disseminated *M. massiliense* in the setting of chronic immunosuppressive therapy (case-patient 7) required >12 procedures including multiple debridements and soft tissue excisions, removal of infected ankle fusion hardware, and osteotomy and arthrotomy of an infected elbow with extensive use of antimicrobial beads and bone cement. A patient with *M. intracellulare* infection disseminated to the left knee and right hip (case-patient 8) required multiple irrigations and debridements, total knee arthroplasty with antimicrobial spacer, and subsequent implantation of a tumor prosthesis. One patient with *M. fortuitum* infection (case-patient 5) had a debridement and eventually required amputation of her hand. One patient had cellulitis and abscess formation caused by *M. marinum* (case-patient 14) requiring a wide excision of the soft tissue around her elbow. One patient with a hip-prosthetic infection caused by *M. abscessus* (case-patient 3) required hemipelvectomy due to the progressive spread of the infection despite >1 year of intravenous therapy, prosthetic hip explantation, and multiple debridements (Table 2).

**Table 2:** Surgical treatment of patients with nontuberculous mycobacterial musculoskeletal infections, Colorado, USA*

Patient no.	Site	Previous surgeries	Symptom onset to first surgery, mo	Surgeries for control of infection at UCH		Time between surgeries, mo	First visit to definitive procedure, mo	Length of follow-up, mo
				First procedure(s)	Definitive procedure			
1	Multiple joints†	Multiple I&Ds, below-knee amputation	UNK	I&D, excision of soft-tissue masses, debridement, synovectomy, amikacin bead implantation	Wide excision of right calf	36	22	7
2	Left foot	Debridement, amputation L 5th toe, nodule excision	2	Debridement of dorsum of left foot	Excisional debridement of left foot	17	14	60
3	Left hip (PJI)	UNK	UNK	Debridement and polyethylene exchange	Hemi-pelvectomy	31	38	14
4	Left thigh	None	18	NA	Above-knee amputation stump revision	NA	14	1
5	Right hand	None	9	NA	Amputation of right hand and forearm	NA	3	37
6	Right hip (PJI)	None	9	Resection arthroplasty, tobramycin cement spacer	Revision THA	4	6	49
7	Multiple joints‡	I&D	1	Hardware removal, I&D, amikacin bead implantation, arthrotomy and osteotomy with amikacin cement, soft-tissue excisions	Excisional debridement of left forearm and right foot with amikacin bead placement§	51	51	2
8	Left knee, right hip	None	UNK	I&D, amikacin cement spacer	TKA with tumor prosthesis, gentamicin cement, amikacin beads	7	8	48
9	Left knee (PJI)	Explant with antimicrobial spacer, I&D	4	I&D, explantation, tobramycin spacer, intramedullary nail with tobramycin cement, revision of spacer	Revision TKA with tobramycin cement and amikacin beads	20	12	20
10	Left knee	Arthroscopic meniscectomy, synovial biopsy, aspirations, arthroscopic synovectomy	2	Radical synovectomy, amikacin bead implantation, resection arthroplasty, knee-spanning intramedullary nail with tobramycin cement	TKA with tumor prosthesis and tobramycin cement	40	12	4
11	Right knee (PJI)	None	12	Explantation, amikacin spacer, I&D, intramedullary nail with amikacin cement	Revision TKA with gentamicin cement and amikacin beads	7	11	17
12	Left knee (PJI)	I&D, polyethylene exchange	0	Explantation, gentamicin cement spacer	Revision TKA with tobramycin cement and amikacin beads	12	5	20
13	Right knee (PJI)	Explant, I&D, antimicrobial spacers	3	Revision TKA, I&D, intramedullary nail with amikacin cement	Revision TKA with amikacin cement and amikacin beads	14	6	1
14	Right elbow	None	8	Wide excision of right forearm, nodule excision	Wide excision of right forearm	4	10	47

*I&D, irrigation and debridement; NA, not applicable; PJI, prosthetic joint infection, THA, total hip arthroplasty; TKA, total knee arthroplasty; UNK, unknown. †Infection was present in patient’s left thigh and knee, and right talus, ankle joint, and subtalar joint. ‡Infection was present in patient’s left elbow, buttock, forearm, calf, right ankle and thigh, and both feet. §Because this patient is in treatment for chronic disseminated infection, definitive procedure is defined as the most recent surgical procedure.

### Histopathology and Microbiology

The species of NTM responsible for the musculoskeletal infection was known or identified in each patient. All patients had tissue specimens taken for culture during their procedure(s) at UCH. Eight patients had microbiologic diagnoses of NTM infection before transfer to UCH, and 6 of these patients were culture-negative at the time of surgery at UCH (case-patients 1, 2, 4, 8, 9, and 13). Antimicrobial therapy was initiated at outside hospitals in 6 of the 8 patients who came to UCH with established microbiologic diagnoses; the other 2 patients were referred directly to infectious diseases specialists at UCH, NJH, or both for treatment. 

Seven patients were infected with a rapidly growing mycobacterial species, whereas the remaining 7 were infected with slowly growing species (Table 1). Ten patients had intraoperative biopsy specimens demonstrating granuloma formation, but only 2 of these had evidence of acid-fast bacilli on direct microscopy. Eight of 14 patients had intraoperative cultures that grew NTM, with speciation consistent with prior data when available. All patients had visual evidence of infection at the time of operation. One patient who had *M. marinum* worked at a pet store that sold fish and aquariums and frequently submerged her hands and arms in the water, and her infection originated at a failed IV site. In the 5 patients with *M. avium* complex, 3 had speciation available that identified *M. intracellulare*.

### Antimicrobial Treatment

All patients were treated with combination antimycobacterial chemotherapy (Table 3) determined on the basis of antimicrobial drug sensitivities (Appendix Table) and recommendations from NJH. Thirteen of 14 patients were receiving antimycobacterial therapy at the time of surgery based on previous culture and drug susceptibility test results. Antimicrobial therapy was not administered around the time of surgery for case-patient 7 because of adverse effects from these medications. 

**Table 3:** Microbiology and antimicrobial treatment of patients with nontuberculous mycobacterial musculoskeletal infections, Colorado, USA*

Patient no.	Species	Oral therapy (duration, mo)	Intravenous therapy (duration, mo)	Total duration of therapy, mo	Time from last surgery to cessation of antimicrobial therapy, mo	Outcome
1	*M. abscessus*	Azithromycin (24)	Tigecycline + amikacin + imipenem (24)	24	0	Cured
2	*M. abscessus*	Azithromycin + linezolid (6)	Amikacin + imipenem (6)	6	0	Cured
3	*M. abscessus*	Azithromycin + clofazimine (20)	Imipenem + amikacin (15)	20	0	Cured
4	*M. abscessus*	Azithromycin + clofazimine (14)	Cefoxitin + amikacin (8)	14	0	Cured
5	*M. fortuitum*	Moxifloxacin (2)	Imipenem (2)	2	1	Cured
6	*M. fortuitum*	Doxycycline + ciprofloxacin + trimethoprim/ sulfamethoxazole (5)	Amikacin (3)	5	3	Cured
7	*M. massiliense*	Azithromycin, linezolid (4)	Cefoxitin, tigecycline, linezolid, amikacin, azithromycin (4)	4†	NA	Treatment failure
8	*M. intracellulare*	Azithromycin + ethambutol + rifabutin (14)	Amikacin (8)	14	1	Cured
9	MAC, speciation unavailable	Azithromycin + clofazimine + ethambutol + rifampin (39)	Amikacin (2)	39	11	Cured
10	*M. intracellulare*	Azithromycin + rifampin + clofazimine + ethambutol (27)	Amikacin (5)	27	7	Cured
11	*M. intracellulare*	Azithromycin + moxifloxacin + ethambutol (9)	None	9	5	Cured
12	MAC, speciation unavailable	Azithromycin + ethambutol + rifampin (7)	None	7	3	Cured
13	*M. gordonae*	Azithromycin + trimethoprim/ sulfamethoxazole + ethambutol (16)	Amikacin (2)	16	2	Cured
14	*M. marinum*	Azithromycin + ciprofloxacin (14)	Imipenem (10)	14	10	Cured

*All bacteria are *Mycobacterium* spp. MAC, *Mycobacterium avium* complex. †Patient elected to discontinue antimicrobial treatment after experiencing intolerable side effects from multiple medications, despite ongoing disseminated infection.

Amikacin-related toxicity occurred in 3 patients. Ototoxicity occurred in 2 patients: case-patient 1 reported hearing loss after 9 months of parenteral amikacin and within 1 month of implantation of amikacin beads, and case-patient 6 experienced tinnitus after 3 months of parenteral amikacin. Kidney injury occurred in case-patient 7 after 3 months of parenteral amikacin and 1 month after implantation of amikacin beads (creatinine level increased from 0.71 to 2.37 mg/dL). Tigecycline was also discontinued in case-patient 7 due to intractable nausea and vomiting within 2 days of initiation, and cefoxitin was discontinued due to fever within 3 days of treatment. Clofazimine also caused intractable nausea and vomiting upon initiation in this patient and was discontinued after 1 month. Apart from case-patient 1, who experienced permanent hearing loss, toxicity resolved with discontinuation of medications. Two patients with *M. avium* complex infection did not receive amikacin due to history of *C. difficile* infection (case-patients 10 and 11). 

The median duration of antimicrobial chemotherapy was 14 months (range 2–39 months). Among the 7 patients with rapidly growing NTM, the median duration was 6 months. The median duration was longer in patients with slow-growing NTM, at 14 months. Twelve patients received injectable antimicrobial drugs as part of their treatment regimen, whereas 2 patients had oral therapy alone. The median duration of therapy with injectable agents was 5 months; rapidly growing strains were treated for a median of 6 months and slow-growing strains for a median of 5 months. The median duration of antimicrobial drug therapy after definitive surgery was 2 months. Patients infected with slow-growing NTM received a more protracted course of postoperative antimicrobial therapy, with a median duration of 5 months, compared with <1 month in patients infected with rapidly growing strains.

### Outcomes

During the follow-up period after surgical intervention (median duration 20 months, range 1–60 months), 13/14 patients did not develop clinical or microbiological relapse and were cured. One (case-patient 7) had clinical and microbiological relapse and was deemed to have failed treatment; the first relapse occurred 2 months after the first surgery to control the infection. The relapse, a disseminated infection distant from the original surgical site, was a likely result of the patient receiving multiple immunosuppressive medications to control systemic lupus erythematosus. She required 12 surgical procedures within the first year and subsequently required ≈1 procedure/year for control of chronic disseminated infection over the next 3 years, primarily in the form of debridement and excision of soft tissue mass. Of note, this patient chose to forgo antimicrobial therapy after the first 4 months of treatment because of intolerable side effects from multiple medications. Two instances of microbiologic relapse were documented for this patient.

## Discussion

We present a series of cases of NTM musculoskeletal infections requiring surgical intervention and antimycobacterial chemotherapy that were managed at UCH and NJH over a 6-year period. NTM musculoskeletal infections have been reported in the literature sporadically due to their rare occurrence; 2 previous case series documented 29 cases over 13 years in South Korea (*27*) and 8 cases of PJI by NTM over 4 decades at the Mayo Clinic in the United States (*26*). In a 6-year period, we have identified 14 case-patients treated at our institutions with unique characteristics, in comparison to previous studies (*25*–*28*). Specifically, the cases we report indicate the importance of a low threshold for suspicion and testing for NTM in any patient who has an unidentified musculoskeletal infection, particularly with evidence of granuloma formation on pathology. Furthermore, for all of our case-patients, NTM infections developed at the site of a prior procedure (prosthetic joint or other intervention), raising the concept of early culture for mycobacteria in conjunction with or after negative results from conventional bacterial culture.

One of the most notable findings from the 3 previous case series studying NTM musculoskeletal infections was that, in all studies, the overwhelming majority of the patients were immunocompetent (*26*–*28*). In contrast, half the patients in our study were immunosuppressed as a result of either an underlying medical condition or a medication used to treat rheumatologic disease. While this may be a manifestation of the relationship between UCH and NJH, our report highlights the importance of hypervigilance for possible NTM in immunocompromised patients with musculoskeletal infections. The most severely immunocompromised patients in our series (case-patients 1, 7, and 8) had disseminated infection. Immunocompromised patients with a history of musculoskeletal surgery should receive a mycobacterial workup as a component of their care.

Another crucial aspect of our case series is the observation that 10/14 cases occurred at the site of a previous musculoskeletal surgery and 6 of these cases involved a joint prosthesis. Moreover, all 14 cases were preceded by an invasive procedure at the initial site of infection. In all but 3 of our surgery-associated cases, the onset of symptoms occurred >3 months after the preceding surgical procedure, suggesting hematogenous spread, possibly due to transient mycobacteremia, with seeding of surgically altered tissue as the likely mechanism of inoculation. This mechanism is particularly likely in case-patients 1, 7, 8, 11, and 12, which comprise 3 cases of disseminated infection in severely immunocompromised patients and 2 late prosthetic joint infections with more than a decade between initial prosthetic joint surgery and eventual infection. The previous report from South Korea noted a minority of periprosthetic infections, but most cases were related to trauma or iatrogenic injections rather than surgery (*27*). 

The report from the Mayo Clinic focused on PJIs but noted only 8 cases over 4 decades (*26*). A recent series of 6 cases of chronic trauma– or intervention-related joint infection not responding to empiric therapy reported no cases of periprosthetic NTM infection (*29*). Our relatively rapid accumulation of surgery-related cases over a 6-year period is due to the relationship between UHC and NJH and NJH’s high volume of NTM cases; we cannot generalize this trend to the general US population, given the unique referral characteristics of our patient population. It is possible that improvements in detection and identification of NTM infections may contribute to this increased case volume, as has been suggested to explain the increasing incidence and prevalence of pulmonary NTM (*30*). Regardless, infections in our study were more likely to occur at the site of a previous orthopedic surgery and in >4 cases led to severe complications requiring partial or full amputation, with 1 patient requiring hemipelvectomy. Three of these patients were infected with *M. abscessus* and 1 with *M. fortuitum*. *M. abscessus* is highly drug resistant and therefore responds poorly to antimicrobial therapy. It is difficult to ascertain if these amputations could have been avoided with earlier diagnosis and aggressive surgical therapy. In several cases, patients with long delays in diagnosis ultimately required amputation; almost one third of patients in our series required amputation.

Given the importance in identifying NTM musculoskeletal infections in the immunocompromised or those with prior surgery, it is unfortunate that our data did not identify more substantial diagnostic criteria based upon inflammatory markers. Whereas the ESR in our patients was usually elevated, the C-reactive protein and leukocyte counts were typically normal. The patient who failed treatment had one of the highest ESRs in our patient group, nearly double the median value of 38 at intake, perhaps because of her chronic autoimmune condition.

All patients had positive mycobacterial cultures at time of initial diagnosis. Thirteen of 14 patients were receiving mycobacterial therapy at the time of surgery at our institution and only half of them were persistently positive for mycobacterial growth despite ongoing symptoms and intraoperative observation of sinus tract formation and suppuration. We are familiar with this phenomenon, and therefore do not require a positive culture to define failure of therapy. If a patient has ongoing signs of infection while receiving effective antimicrobial drugs (based on drug sensitivity testing), then they need more aggressive resection of infected tissue. The 3 patients with PJI and rapidly growing NTM infection all required prolonged courses of IV and oral antimicrobial therapy. One of the 3 patients with *M. avium* complex–related PJI also required 2 months of concomitant IV amikacin. All patients with PJIs required explantation of the infected hardware followed by antimicrobial spacer placement for >3 months in addition to ongoing postoperative oral or intravenous antimicrobial therapy to achieve a successful outcome. In >5 cases, microbiologic diagnosis of mycobacterial infection was only confirmed during the initial surgical intervention, and in each of these cases, prior or subsequent surgeries were required for ultimate control of infection. Ultimately, the fact that 10/14 cases required multiple surgeries for control of infection with long intervals from onset of symptoms and initial seeking of care to identification of the infectious agent and finally definitive surgical treatment underscores the need for early identification and aggressive treatment of NTM infection.

Appendix Additional information about nontuberculous mycobacterial musculoskeletal infections, Colorado, USA.

## References

[1] Prevots DR, Shaw PA, Strickland D, Jackson LA, Raebel MA, Blosky MA, et al. Nontuberculous mycobacterial lung disease prevalence at four integrated health care delivery systems. Am J Respir Crit Care Med. 2010;182:970–6. 10.1164/rccm.201002-0310OC 20538958 PMC2970866

[2] Adjemian J, Olivier KN, Seitz AE, Holland SM, Prevots DR. Prevalence of nontuberculous mycobacterial lung disease in U.S. Medicare beneficiaries. Am J Respir Crit Care Med. 2012;185:881–6. 10.1164/rccm.201111-2016OC 22312016 PMC3360574

[3] Al-Houqani M, Jamieson F, Mehta M, Chedore P, May K, Marras TK. Aging, COPD, and other risk factors do not explain the increased prevalence of pulmonary *Mycobacterium avium* complex in Ontario. Chest. 2012;141:190–7. 10.1378/chest.11-0089 21724552

[4] Spaulding AB, Lai YL, Zelazny AM, Olivier KN, Kadri SS, Prevots DR, et al. Geographic distribution of nontuberculous mycobacterial species identified among clinical isolates in the United States, 2009–2013. Ann Am Thorac Soc. 2017;14:1655–61. 10.1513/AnnalsATS.201611-860OC 28817307 PMC5711271

[5] Griffith DE, Aksamit T, Brown-Elliott BA, Catanzaro A, Daley C, Gordin F, et al.; ATS Mycobacterial Diseases Subcommittee; American Thoracic Society; Infectious Disease Society of America. An official ATS/IDSA statement: diagnosis, treatment, and prevention of nontuberculous mycobacterial diseases. Am J Respir Crit Care Med. 2007;175:367–416. 10.1164/rccm.200604-571ST 17277290

[6] Smith GS, Ghio AJ, Stout JE, Messier KP, Hudgens EE, Murphy MS, et al. Epidemiology of nontuberculous mycobacteria isolations among central North Carolina residents, 2006-2010. J Infect. 2016;72:678–86. 10.1016/j.jinf.2016.03.008 26997636

[7] Cassidy PM, Hedberg K, Saulson A, McNelly E, Winthrop KL. Nontuberculous mycobacterial disease prevalence and risk factors: a changing epidemiology. Clin Infect Dis. 2009;49:e124–9. 10.1086/648443 19911942

[8] Booth JE, Jacobson JA, Kurrus TA, Edwards TW. Infection of prosthetic arthroplasty by *Mycobacterium fortuitum.* Two case reports. J Bone Joint Surg Am. 1979;61:300–2. 10.2106/00004623-197961020-00029 422622

[9] Horadam VW, Smilack JD, Smith EC. *Mycobacterium fortuitum* infection after total hip replacement. South Med J. 1982;75:244–6. 10.1097/00007611-198202000-00036 7058374

[10] Heathcock R, Dave J, Yates MD. *Mycobacterium chelonae* hip infection. J Infect. 1994;28:104–5. 10.1016/S0163-4453(94)94533-0 8163823

[11] Pring M, Eckhoff DG. *Mycobacterium chelonae* infection following a total knee arthroplasty. J Arthroplasty. 1996;11:115–6. 10.1016/S0883-5403(96)80170-7 8676110

[12] Luque AE, Kaminski D, Reichman R, Hardy D; Amneris E. Luque, Dorothy Kaminski. *Mycobacterium szulgai* osteomyelitis in an AIDS patient. Scand J Infect Dis. 1998;30:88–91. 10.1080/003655498750002376 9670366

[13] Breda L, de Michele G, Nozzi M, De Sanctis S, Di Marzio D, Chiarelli F. Non-tuberculous mycobacterial osteomyelitis: an unusual cause of hip pain in immunocompetent children. Rheumatol Int. 2009;29:1487–9. 10.1007/s00296-009-0844-4 19156420

[14] Wang S-X, Yang C-J, Chen Y-C, Lay C-J, Tsai C-C. Septic arthritis caused by *Mycobacterium fortuitum* and *Mycobacterium abscessus* in a prosthetic knee joint: case report and review of literature. Intern Med. 2011;50:2227–32. 10.2169/internalmedicine.50.5610 21963746

[15] Garcia DC, Sandoval-Sus J, Razzaq K, Young L. Vertebral osteomyelitis caused by *Mycobacterium abscessus.* BMJ Case Rep. 2013;2013(aug07 1):cr2013009597. 10.1136/bcr-2013-009597 23925676 PMC3761664

[16] Suy F, Carricajo A, Grattard F, Cazorla C, Denis C, Girardin P, et al. Infection due to *Mycobacterium thermoresistibile*: a case associated with an orthopedic device. J Clin Microbiol. 2013;51:3154–6. 10.1128/JCM.00925-13 23843483 PMC3754661

[17] Bakhsh WR, Mesfin A. *Mycobacterium kansasii* infection of the spine in a patient with sarcoidosis: a case report and literature review. J Surg Orthop Adv. 2014;23:162–5. 10.3113/JSOA.2014.0162 25153815

[18] Hazara AM, Edey M, Roy A, Bhandari S. Rapidly growing non-tuberculous mycobacterial infection in a renal transplant patient after alemtuzumab induction. Transpl Infect Dis. 2014;16:847–52. 10.1111/tid.12269 25040696

[19] Kato S, Murakami H, Demura S, Yoshioka K, Hayashi H, Yokogawa N, et al. Vertebral osteomyelitis caused by *Mycobacterium abscessus* surgically treated using antibacterial iodine-supported instrumentation. Case Rep Orthop. 2014;2014:197061. 10.1155/2014/197061 25544922 PMC4269211

[20] Nguyen HH, Fadul N, Ashraf MS, Siraj DS. Osteomyelitis infection of *Mycobacterium marinum:* a case report and literature review. Case Rep Infect Dis. 2015;2015:905920. 10.1155/2015/905920 25664190 PMC4312609

[21] Sharma VK, Pai G, Deswarte C, Lodha R, Singh S, Kang LW, et al. Disseminated *Mycobacterium avium* complex infection in a child with partial dominant interferon gamma receptor 1 deficiency in India. J Clin Immunol. 2015;35:459–62. 10.1007/s10875-015-0173-1 26054576

[22] Silva JT, López-Medrano F, Fernández-Ruiz M, San-Juan R, Ruiz-Cano MJ, Delgado JF, et al. *Mycobacterium abscessus* pulmonary infection complicated with vertebral osteomyelitis in a heart transplant recipient: case report and literature review. Transpl Infect Dis. 2015;17:418–23. 10.1111/tid.12381 25816889

[23] Wood BR, Buitrago MO, Patel S, Hachey DH, Haneuse S, Harrington RD. *Mycobacterium avium* complex osteomyelitis in persons with human immunodeficiency virus: case series and literature review. Open Forum Infect Dis. 2015;2:ofv090. 10.1093/ofid/ofv090 26180837 PMC4499669

[24] Kuntz M, Seidl M, Henneke P. Osteomyelitis because of *Mycobacterium xenopi* in an immunocompetent child. Pediatr Infect Dis J. 2016;35:110–3. 26418244 10.1097/INF.0000000000000933

[25] Marchevsky AM, Damsker B, Green S, Tepper S. The clinicopathological spectrum of non-tuberculous mycobacterial osteoarticular infections. J Bone Joint Surg Am. 1985;67:925–9. 10.2106/00004623-198567060-00015 4019542

[26] Eid AJ, Berbari EF, Sia IG, Wengenack NL, Osmon DR, Razonable RR. Prosthetic joint infection due to rapidly growing mycobacteria: report of 8 cases and review of the literature. Clin Infect Dis. 2007;45:687–94. 10.1086/520982 17712751

[27] Park JW, Kim YS, Yoon JO, Kim JS, Chang JS, Kim JM, et al. Non-tuberculous mycobacterial infection of the musculoskeletal system: pattern of infection and efficacy of combined surgical/antimicrobial treatment. Bone Joint J. 2014;96-B:1561–5. 10.1302/0301-620X.96B11.33427 25371475

[28] Johnson MG, Stout JE. Twenty-eight cases of *Mycobacterium marinum* infection: retrospective case series and literature review. Infection. 2015;43:655–62. 10.1007/s15010-015-0776-8 25869820 PMC6535045

[29] Gundavda MK, Patil HG, Agashe VM, Soman R, Rodriques C, Deshpande RB. Nontuberculous mycobacterial infection of the musculoskeletal system in immunocompetent hosts. Indian J Orthop. 2017;51:205–12. 10.4103/0019-5413.201718 28400668 PMC5361473

[30] Johnson MM, Odell JA. Nontuberculous mycobacterial pulmonary infections. J Thorac Dis. 2014;6:210–20. 24624285 10.3978/j.issn.2072-1439.2013.12.24 PMC3949190

